# Number comparison in bilinguals is not affected by their second language

**DOI:** 10.1038/s41598-026-52236-w

**Published:** 2026-07-10

**Authors:** Silke M. Göbel, Veniamin Shiron, Miriam Tucker, Angela De Bruin

**Affiliations:** 1 Department of Psychology, University of York, York, UK; 2 Centre for Research in Equality in Education (CREATE), University of Oslo, Oslo, Norway

**Keywords:** Unit-decade compatibility effect, Number word inversion, Language switching, L2, Bilingualism, Language and linguistics, Language and linguistics, Neuroscience, Psychology, Psychology

## Abstract

Language background can show subtle effects on number comparison even with Arabic digits. Number pairs can be decade-unit compatible, i.e. the decision about number size is the same for unit and decade (e.g. 24 and 57, 2 < 5 and 4 < 7), or decade-unit incompatible (e.g. 29 and 57, 2 < 5 but 9 > 7). Incompatible pairs often lead to longer response times. This compatibility effect is larger in languages with number word inversion (e.g. German). We aimed to investigate the influence of language on the compatibility effect in bilinguals. In Experiment 1, we compared the compatibility effect in German monolinguals and German-English bilinguals. We found a significant compatibility effect, but no significant differences between the mono- and bilinguals. In Experiment 2, we manipulated language activation within participants by asking German-English bilinguals to describe scenes either in English or German before completing number comparison trials. There was a significant cost of switching languages, showing that participants did activate German and English. However, the size of the compatibility effect was not significantly influenced by participants activating German or English. Overall, we did not find an influence of a bilingual’s language on number comparison with Arabic digits.

## Which language matters? Number comparison in bilinguals

Numbers are ubiquitous in modern life. We encounter exact numbers in different formats, most frequently as spoken or written number words (‘twenty-four’) or Arabic digits (24). While number words are language-specific^1^, Arabic digits are often seen as an abstract, pure and non-linguistic number format. However, even basic number processing of Arabic digits is influenced by the language background of the person processing them^2,3^.

Strong support for an influence of language background on number processing comes from developmental research^4–9^. For example, whether a language has a clear singular/plural distinction (one frog, two frog***s***, three frog***s***, four frog***s***) might influence the acquisition of the meaning of the number word ‘one’^10^. English-speaking children typically learn the meaning of ‘one’ around 24 to 26 months^10,11^. But in languages with less consistent or without obligatory associations of number words with singular/plural nouns, such as Japanese, children learn the meaning of the number word ‘one’ several months later^10,12,13^ than in English.

Moving on to larger number words, number word construction is entirely regular in some languages, such as Japanese and Mandarin. Once children have learnt the number words one to ten in these languages, they can combine these ten elements to create all number words up to 99. Several studies investigated the acquisition of teen numbers (11–19) in different languages and reported a delay in number word acquisition for numbers larger than ten for languages for which number words are less regular^14–17^.

One’s language acquired from birth does not only influence number word acquisition and numerical and arithmetic development^4,18–22^, it also affects number processing of Arabic digits in adults^2,22–24^. One effect that has often been used to investigate language influences on number processing in adults is the unit-decade compatibility effect^25^. This effect is observed in multi-digit number comparison, when participants are asked to choose, for example, the numerically larger number out of two-digit number pairs in Arabic format. A number pair is considered *unit-decade compatible* when comparing tens and units leads to similar response biases (e.g., 42_57: 4 < 5 and 2 < 7). In turn, a number pair is *unit-decade incompatible* when the respective comparisons lead to opposite response biases (e.g., 47_62: 4 < 6 but 7 > 2). Compatible number pairs are usually responded to faster and with fewer errors than incompatible number pairs^25–27^.

Several studies have shown that the size of the compatibility effect is influenced by which language people speak^2,28,29^. Comparing for example the compatibility effect in a German- and an English-speaking sample^28^, a more pronounced effect was found in the German-speaking group. The authors explain this finding by differences in the verbal number word systems. While the English number word system is fairly consistent for numbers larger than 20, German two-digit number words are inverted with respect to the Arabic digit notation (42 is spoken as ‘zweiundvierzig’, literally translated ‘two-and-forty’). That means that the unit digit is named first in German in contrast to the Arabic format. Clearly, mapping between number words and Arabic digits is harder in languages with number word inversion^24^. Cross-linguistic differences in the compatibility effect can be subtle, for example the compatibility effect has been found to be significant by participants but not by items^28^ or was absent in mean reaction times, but present when z-scores were used^29^. Although the difference between groups might be small, a larger compatibility effect in German than English speakers^28^ demonstrates that even adults activate number words during processing of numbers in Arabic format.

Most of the previous research in this area has focused on different language groups. However, over half of the world’s population is bi- or multilingual^30^and many of these bilinguals need to alternate between different number-word systems in their everyday communications. The two languages of a bilingual influence each other and compete with each other for selection (e.g.^31^,), but knowledge about how a bilingual’s languages influence number processing in adults is still limited.

For exact arithmetic, language of instruction/training seems to matter for bilinguals^32^. For example, bilinguals trained in exact arithmetic were faster when they were tested in the language they were originally trained in than when tested in their other language^33^(see also^34)^. In terms of more general language effects (beyond the language of training and testing), a meta-analysis^35^ found that bilinguals were faster and more accurate in number naming and arithmetic in their first (L1) than second (L2) language. Schiltz et al. (2024)^3^ in a recent review of the influence of multilingualism on numerical and mathematical competencies also conclude that performance in basic numerical skills is better in L1 (and/or in the language of mathematics learning) than in L2.

These findings are in line with a prominent model of number processing, the Triple Code Model^36^. This model proposes the existence of three independent but interconnected numerical codes: a language-dependent verbal code for spoken and written number words, an analog magnitude code representing numerical magnitude approximately and a visual code for numerical symbols such as Arabic digits. The verbal code is believed to be essential for retrieving arithmetic facts, and the language-independent analog magnitude code for number comparison and estimation. When numbers are presented visually as Arabic digits, they are first processed by the visual code. Then there are two processing routes, a direct asemantic route between the visual and the approximate code for number comparison and an indirect route which goes from the visual code via the verbal code to activate the approximate code. The influence of language during number comparison of Arabic digits along with other findings^28,29^ suggests that both routes are activated in parallel when processing Arabic digits.

**Fig. 1:**
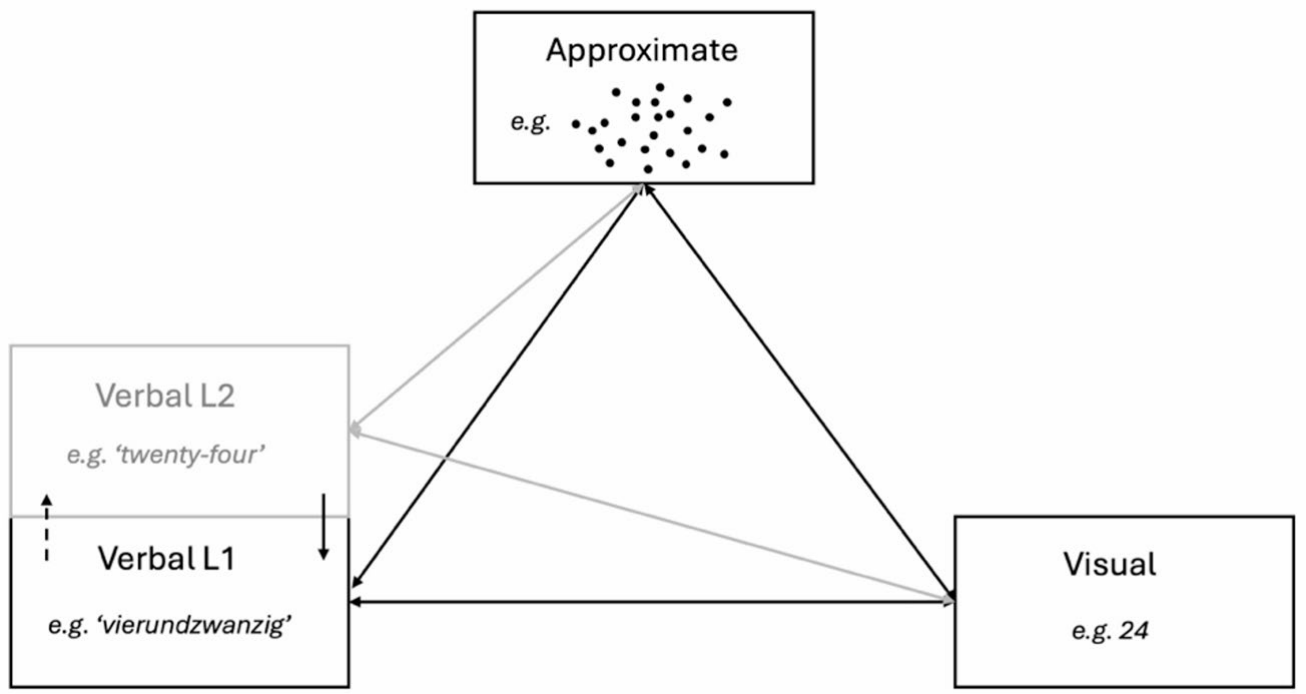
Bilingual triple code model (BTCM) adapted from Lachelin et al. (2024).

Lachelin et al. (2024)^37^ have recently suggested a bilingual Triple code model (BTCM). In this model, bilinguals have two language-specific codes for number processing (see Fig. 1), a verbal code in L1 and a verbal code in L2, which are connected and can influence each other. The presence of two language-specific nodes raises the question which language (L1 or L2) bilinguals activate most during number processing of Arabic digits.

To our knowledge, only two previous studies investigated the compatibility effect with Arabic digits in bilinguals. Van Rinsveld et al. (2016)^38^ investigated the compatibility effect in German or French monolinguals and German-French bilinguals and found that the German-French bilinguals exhibited the same pattern of results as the German monolinguals. However, their bilingual group was quite different from the two monolingual groups. The bilingual participants’ L1 was Luxembourgish (which developed from a dialectal variant of German) and they also spoke German and French, which are official primary school languages for numeracy and literacy in Luxembourg. German monolinguals were University students at the University of Graz in Austria and French monolinguals were from Brussels in Belgium.

Macizo et al. (2011)^39^ tested whether the level of L2 proficiency affected the size of the compatibility effect. Two groups of German(L1)-English(L2) bilinguals performed a double-digit number comparison task with Arabic digits, L1 and L2 number words. Participants were equated in terms of their proficiency in German (L1), but were either more or less proficient in English (L2; based on self reports only). The size of the compatibility effect for Arabic digits was not affected by L2 proficiency, while proficiency affected the size of the compatibility effect with number words. While both studies provide evidence for little influence of L2 on the compatibility effect, this could be due to the specific language background of their participants. Macizo et al. focused on proficiency assessed through self reports, which are not always accurate reflections of objective proficiency^40^, and the German monolingual and bilingual groups in Van Rinsveld et al.’s (2016)^38^ study had some significant differences in terms of first language, language of instruction, and language environment (e.g., early exposure to German in the bilingual group growing up in a very multilingual environment).

### Current study

The aim of our study was to investigate the effect of a bilingual’s second language (compared to functional monolinguals, Experiment 1; compared to the bilingual’s first language, Experiment 2) on the compatibility effect.

We examined this by comparing German-functional monolinguals and German-English bilinguals in Experiment 1 and by manipulating language activation within German-English bilinguals in Experiment 2. Larger compatibility effects have been reported in participants with an L1 with number word inversion. Across the two experiments, we therefore examined the effect on the compatibility effect of an L2 without number word inversion (English) relative to an L1 with number word inversion (German).

In both studies we expected to find an overall significant unit-decade compatibility effect (significantly longer response times for incompatible than compatible number pairs). For Experiment 1, based on previous research showing a larger compatibility effect in German- than English-speaking participants^2,28,29^, we predicted a reduced compatibility effect in our German-English bilingual speakers compared to the German monolinguals. For Experiment 2, we expected a larger compatibility effect for number comparison trials following activation of German (elicited through a preceding scene description trial) than following activation of English. (Please note, that, due to an oversight, this hypothesis was stated incorrectly in the opposite direction in our pre-registration).

## Experiment 1

### Methods

#### Participants

Experiment 1 was completed by 31 German functional monolinguals and 30 German-English bilinguals. While we will refer to the German “functional monolingual” group as “monolingual”, these participants did have some L2-English knowledge. However, they were functional monolinguals in the sense of predominantly or only using their L1-German in their daily lives. The two groups did not differ significantly in terms of age (monolingual *M age* = 29.1 years old, *SD* = 6.3; bilingual *M age* = 32.0 years old, *SD* = 6.8; *t*(59) = 1.732, *p* =.089). The monolingual group included fewer female participants than the bilingual group (monolingual 13 female, 18 male; 23 right-handed; bilingual 21 female, 8 male, 1 transgender; 25 right-handed). Participant groups did not differ significantly in terms of mean number of years of education (monolingual *M* = 14.8 years, *SD* = 4.5; bilingual *M* = 16.5, *SD* = 3.7; *t*(59) = 1.593, *p* =.116); but bilinguals overall had a higher level of completed education than the monolingual group (monolingual: 17 secondary school/A-levels/vocational training; 10 undergraduate degree; 3 postgraduate degree; 1 doctorate; bilingual: 9 secondary school/A-levels; 6 undergraduate degree; 9 postgraduate degree; 6 doctorate; Χ^2^(3) = 10.019, *p* =.018). All participants provided informed consent at the start of the study and ethics approval was granted by the Ethics Committee in the Department of Psychology at the University of York. The study was *pre-registered*https://osf.io/52d9t and was conducted following the Declaration of Helsinki.

Before completing the main study, participants completed a pre-screening. This was completed by 71 potential monolingual and 62 potential bilingual participants. We only invited participants to take part in the main study if they met the following background criteria. All participants had to have no (self-reported) neurological, language, or reading difficulties, or vision or hearing impairments. All participants had to have acquired German from birth, with no other languages acquired from birth and spoken fluently (apart from English in the bilingual group). All participants also had to score at least 60% on the German LexTALE, a short lexical decision task included to measure vocabulary knowledge. In this task, participants had to indicate if letter strings formed an existing word or not^41^. LexTALE scores were calculated following the recommended scoring^41^: ((number of words correct/total words * 100) + (number of nonwords correct/total nonwords *100))/2.

For the functional monolinguals, our additional language requirements were as follows: no English acquisition from birth and weekly use of English being reported as “sometimes” or lower. All included participants also reported having learnt numbers during childhood in German and not having used numbers in school and/or university, or in their current lives, in a language other than German. All monolingual participants were born in Germany or Austria and were currently living there. Given that English is taught in schools in Germany and Austria, we could not recruit people without any knowledge of English. We therefore focused on “functional monolinguals” who do not use English more than sometimes in their daily lives. These participants did have some L2-English knowledge (see Table 1). The majority of participants reported a self-rated proficiency below 8/10 for English speaking and understanding. Although our pre-registered cut-off was < 8/10, we also included three participants who self-rated their English understanding (and in one case English speaking) as 8/10. This was done to not reduce our sample size further. Of the 71 monolingual participants who completed the pre-screening, 37 were invited for and also completed the main study. Of those, one participant was excluded afterwards as they reported speaking another language well and at home from childhood, one participant because they reported frequent use of English, and four participants because they completed the pre-screening in the same session as the main task but did not actually meet the requirements due to using English too frequently and having a self-rated English proficiency of 9/10. This left us with 31 monolingual participants in the analysis.

For the bilinguals, our pre-registered inclusion criteria were as follows: did not use another language than German or English at home or school during childhood; self-rated English proficiency at least 8/10 for speaking and understanding; uses English at least “frequently” on a weekly basis; current language use score on the Language and Social Background Questionnaire (LSBQ)^42^ of 2.5 or higher (1 = all German use; 5 = all English use); and no indication that another language than German or English was used exclusively in any of the contexts assessed. To examine this, we used a modified version of the LSBQ, asking about participants’ language use in different contexts, activities, and with various conversation partners. All participants also scored at least 60% on the LexTALE in both English and German. We had an additional pre-registered point stating we would exclude participants who reported their language use during childhood to be above 2.5 on the LSBQ (where higher scores reflect more English use). Two participants scored slightly above this but were included because this only reflected some English education as a teenager. As can be seen in Table 1, bilingual participants had a high proficiency in and frequent use of both languages. They also reported occasional to regular language switching (on a scale from 1 = never to 10 = very frequently) on a daily basis (*M* = 6.3, *SD* = 2.7), within a conversation (*M* = 4.8, *SD* = 2.4), and within a sentence (*M* = 4.3, *SD* = 2.7).

In terms of proficiency (see Table 1), participants in the bilingual group showed significantly higher self-rated English proficiency than the monolingual group (all *p*s < 0.05). German proficiency scores showed no group differences in the self ratings or LexTALE (*p*[min] = 0.192, *p*[max] = 0.999), with the exception of self-rated German speaking proficiency being slightly lower in the bilingual group (*p* =.031).

All bilingual participants learnt numbers in German during childhood and, where relevant, used German and/or English for numbers at university and when using numbers in their daily lives, with no use of other languages. In the final questionnaire, we only asked participants to choose one language that they predominantly used for number tasks (e.g., subtractions) and remembering numbers. We did not examine how frequently participants encountered numbers in English or in German and could therefore not assess our pre-registered point of encountering English numbers at least 25% of the time. While all participants were born in Germany, they were currently living in an English-dominant country (e.g., UK or US), making it very likely that they had regular exposure to numbers in English.

**Table 1:** Participants’ language background in terms of age of acquisition, self-rated proficiency, vocabulary assessed through the LexTALE, and language use.

	Functional monolinguals	Bilinguals
*N*	31	30
**Age of Acquisition (in years)**		
German	0.0 (0)	0.0 (0)
English	X	8.4 (3.0)
**Self-rated German proficiency (1–10)**		
Speaking	9.9 (0.4)	9.6 (0.7)
Understanding	10.0 (0.0)	10.0 (0.0)
Reading	9.9 (0.2)	9.9 (0.3)
Writing	9.8 (0.6)	9.6 (0.6)
**Self-rated English proficiency (1–10)**		
Speaking	4.5 (1.7)	9.1 (0.8)
Understanding	5.4 (1.8)	9.4 (0.8)
Reading	5.3 (2.0)	9.3 (0.8)
Writing	4.4 (1.9)	9.1 (0.9)
**LexTALE (0–100%)**		
German	88.4 (6.3)	88.6 (7.1)
English	X	90.7 (7.3)
**Language use**		
***L2-English***		
Never	5 people	0
Rarely	12 people	0
Sometimes	14 people	0
Frequently	0	4 people
Always	0	26 people
**Childhood LSBQ (1 = all German**,** 5 = all English)**	X	1.6 (0.5)
**Current LSBQ (1 = all German**,** 5 = all English)**	X	4.0 (0.3)

Note: The standard deviation is reported in brackets.

Participants were also asked questions about their experience with numbers and mathematics, as reported in Table 2. The two participant groups were comparable in terms of the number of hours they had received mathematics teaching in school, the number of people studying mathematics at university level, and the number of people working with numbers in their jobs. Both groups also learnt mathematics in German in school and were taught numbers in German as their first language. The two groups differed, as intended, in their current use of English for talking about mathematics in university, their main language for using numbers for daily activities, and remembering number sequences. These tasks were all performed in German in the monolingual group while within the bilingual group, approximately half of the participants reported English to be their main language for number related activities.

**Table 2:** Participants’ mathematics education and language use for number/mathematics related topics.

	Functional monolinguals	Bilinguals
*N*	31	30
Number of mathematics education hours during early school years (number of mathematics hours during high school in parentheses)		
1–2 h per week	3 (0)	0 (0)
2–3 h per week	9 (4)	10 (5)
3–5 h per week	15 (21)	20 (21)
5 + hours per week	4 (6)	0 (4)
Language of mathematics education early years	All German	1 English, 29 German
Language of mathematics education high school	All German	2 English, 28 German
Number of people who studied other mathematics related subjects at school	11	12
Number of people who studied a mathematics related degree at university level	5	5
Language used when talking about mathematics related topics at university	23 German (rest not relevant)	10 German, 13 English (rest not relevant)
First language to learn numbers	All German	All German
Number of people working with numbers in current job	17	15
Main language when using numbers in daily life (like subtractions)	All German	13 German; 17 English
Main language when remembering number sequences, like phone numbers	All German	15 German; 15 English

Our pre-registered plan was to also include a comparison group of German-Dutch bilinguals. However, recruitment was very difficult and only ten participants started the study and did not always meet our inclusion criteria. We therefore decided to only include German functional monolinguals and German-English bilinguals in this experiment, which still allowed us to answer our main research question regarding the influence of English in bilinguals. Our sample sizes for these two groups were slightly lower than the initial pre-registered aim of 40 participants per group. Because we faced difficulties recruiting German-speaking monolinguals, we decided to follow our pre-registered stopping rule of 30 participants.

Our power analysis (see pre-registration) was based on previous research comparing the compatibility effect between German- and English-speaking adults showing a partial eta squared effect size of 0.11^29^. Using G*Power, our power analysis showed 95% power with 34 participants per group, using an effect size of 0.15. (However, as pointed out by a reviewer, rather than basing the expected effect size on comparisons between monolingual groups, it could be argued that a smaller effect size would be expected when comparing a monolingual German-speaking group to a German-English bilingual group.)

### Design

The number-comparison task had two independent variables: Type (compatible, e.g., 46 − 21, or incompatible, e.g., 81 − 32; within-subject) and Group (monolingual or bilingual; between-subject). Additional filler items (20%, within-decade comparisons, e.g., 68 − 61) were included in the task but not in the analysis. Our dependent variable was reaction times (RTs).

### Procedure

Participants were recruited through Prolific.co and tested using Gorilla.sc^43^. All instructions throughout the study were presented in German. Most participants (all bilinguals and all apart from 8 monolinguals) first completed a pre-screening, which included our questionnaire and the LexTALE(s). Only participants meeting the inclusion criteria (see “Participants”) were invited to also take part in the main study. For a few monolinguals, we combined the pre-screening and main study to facilitate recruitment.

In the main study, participants were asked to complete a two-digit number magnitude comparison task. In each trial, two numbers were presented simultaneously and above each other in the center of the screen. The stimulus set consisted of 300 two-digit Arabic number pairs between 21 and 98, with 120 unit-decade compatible (e.g., 43_58), 120 unit-decade incompatible (e.g., 48_53; taken from^25^, and 60 within-decade filler pairs (e.g., 51_56, 20% of all trials). Overall distance, decade distance, unit distance, and problem size (i.e., the sum of the two to-be-compared numbers) were matched between the unit-decade compatible and unit-decade incompatible stimulus groups.

Participants had to choose the larger of two numbers presented. They were instructed to respond as quickly and accurately as possible with the right index finger (pressing the U key) in case the upper number was larger, and with the left index finger (pressing the N key) when the bottom number was larger. For half of the pairs, the upper number was larger. In the other half, the lower number was larger. Participants had to press the same button not more than twice in a row. Stimuli were presented in white (font: Courier New, bold; font size: 24) against a black background until a response was given. Preceding each trial, a fixation cross was presented in the middle of the screen for 500 ms. Trials were separated by an interstimulus interval of 500 ms. If no response was given, the next trial was started automatically after three seconds.

The experiment started with 12 practice trials with feedback, followed by five experimental blocks of 60 number pairs each without feedback.

### Data analysis

The task data are available at https://osf.io/gjnwt/.

The RT data were analysed using ANOVAs and linear mixed-effects (LME) analyses (using R version 4.4.1, lme4 version 1.1–36 and lmerTest version 3.1-3.1)^44,45^. Both types of analyses were pre-registered. As this was a student-led project, we included the use of ANOVAs, but we added the use of LMEs to capture item-level variability too. Accuracy was high (*M* = 97.2%, *SD* = 2.4 on compatible and incompatible trials combined; all participants scored 90% or higher) and therefore not analysed further. Prior to RT analysis, we removed inaccurate responses and RTs > 3 *SD* above or below the mean per participant and condition (0.8% of correct trials)^46^. No RTs were below 200ms. Visual inspection of the RT data revealed a non-normal distribution and RTs were therefore log-transformed. The analysis only included compatible and incompatible trials, fillers (within-decade comparisons) were not included. The LME analysis included Type (compatible coded as −0.5 and incompatible as 0.5) and Group (monolingual coded as −0.5 and bilingual coded as 0.5) as fixed effects. Participant and item (number pair shown on the screen) were included as random effects, with the corresponding within-participant and within-item slopes. As pre-registered, we started with a maximal structure (following e.g., Barr et al., 2013^47^; Brauer & Curtin, 2018^48^; but cf. Matuschek et al., 2017^49^, for alternative approaches). The full model converged. We also ran the corresponding ANOVA and two non-pre-registered Bayesian analyses. One was a Bayesian mixed regression model, including the same fixed and random effects as the LME analysis. We ran Bayesian linear mixed models with the brms package (version 2.22.0). We assumed normally distributed residuals and an improper flat prior over all real numbers for fixed effects (the package’s default) and estimated the models using Markov Chain Monte Carlo simulations with 4 chains for 100,000 iterations. Below we report the mean point estimate and 95% credible interval. Credible intervals including zero can be interpreted as 95% probability that the true value is within the interval including zero (i.e., no difference in number-compatibility effect between groups). Rhat values were 1.0 and bulk and tail-effective sample size above 10,000. We also used a Bayesian ANOVA to quantify evidence for the null versus the alternative hypothesis (using the BayesFactor package, version 0.9.12–4.7^51^). Here, we compared a null model including main effects of Type (compatibility effect) and Group to a model including an interaction between Type and Group to assess evidence for/against a difference in compatibility effect between bilinguals and monolinguals. The Bayes Factor (BF) is reported in the form BF_01_ (evidence for the null over the alternative hypothesis), with values above 1 supporting the null hypothesis. We used the BayesFactor package’s default distribution parameters (cf. Rouder et al., 2012^52^, using a Cauchy distribution with a scale parameter of sqrt(2)/2). Note that while we pre-registered the use of z-scored RTs in the ANOVA, we used log-transformations instead for consistency with the LME analysis. Z-scoring of RTs was furthermore not needed given that no overall RT differences were observed between the two groups.

## Results and discussion

**Fig. 2:**
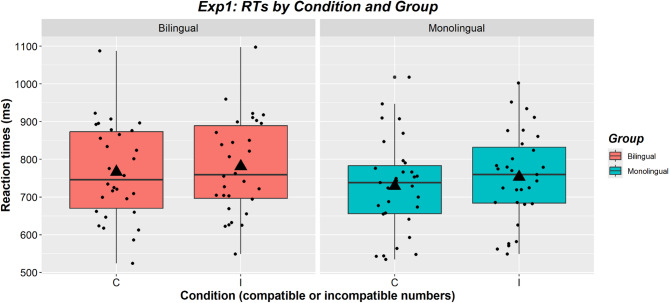
Mean RTs by Type (the condition being Compatible or Incompatible numbers) and Group (bilingual and monolingual). The centre of the triangle depicts the group mean, the black horizontal line the median. Black dots represent individual participants.

The LME analysis showed a significant effect of Type (β = 0.027, *SE* = 0.013, *t* = 2.088, *p* =.038), showing that there was a compatibility effect with faster responses to compatible number pairs (*M* = 748ms, *SD* = 125ms) than to incompatible pairs (*M* = 767ms, *SD* = 123ms), see Fig. 2. There was no main effect of Group (β = 0.045, *SE* = 0.041, *t* = 1.094, *p* =.279), suggesting bilinguals and monolinguals responded at comparable speed overall (Monolingual *M* = 741ms, *SD* = 121ms; Bilingual *M* = 774ms, SD = 126ms). The number compatibility effect was numerically a little smaller for bilinguals (*M* = 14ms, *SD* = 18ms) than for monolinguals (*M* = 24ms, *SD* = 32ms) but this did not reach significance (β = −0.014, *SE* = 0.008, *t* = −1.843, *p* =.070). The Bayesian model showed a point estimate for the interaction of −0.01, with a 95% credible interval that included 0: [−0.03, 0.00].

The ANOVA showed the same patterns, with a significant main effect of Type (*F*(1,59) = 49.220, *p* <.001, ηp² = 0.455), no significant effect of Group (*F*(1,59) = 1.215, *p* =.275, ηp² = 0.020), and no significant interaction between Group and Type (*F*(1,59) = 3.390, *p* =.071, ηp² = 0.054). The Bayesian analysis provided no support for the presence, or absence, of a compatibility-effect difference between the two groups (BF_01_ = 1.72, error% = 2.57).

All instructions were provided in German, thereby creating a German context throughout the study for all participants. Within the bilingual group, however, some participants reported predominantly using English for daily-life number processing (for tasks such as subtractions or for remembering numbers), while others reported using German, or did not report a consistent preference across different types of number activities. An exploratory analysis comparing 12 participants reporting daily-life number processing in English to 10 participants reporting doing such processing in German showed a slightly smaller number compatibility-effect cost in the task for participants more likely to use English to process numbers (*M* cost = 8ms, *SD* = 21ms) than those more likely to use German (*M* cost = 20ms, *SD* = 12ms; *t*(20) = 1.449, *p* =.163; *d* = 0.621).

Experiment 1 showed no significant difference between the bilingual and monolingual groups. We attempted to make our functional monolingual group as monolingual as possible, by only including participants who predominantly use German in their daily lives, including for number-related tasks. In contrast, our bilingual participants were living in an English-dominant society and reported frequent use of English for daily-life interactions. However, if any potential effects of language use are small, they might be less likely to arise in these group comparisons, also considering the relatively small size of the number-compatibility effect. The groups tested differed in some ways, including their highest level of education completed. This could have influenced number processing and could have masked any language-related effects, if they exist. Furthermore, our monolingual participants were functional monolinguals who only used their L1, but still had some proficiency in their L2-English. This was inevitable given the presence of English in Germany and Austria, and the compulsory English classes children receive at school. While it is unlikely these participants use English for number processing (given that they learnt to do this in German and only rarely use English in general), we cannot rule out some influence of L2-English processing in our task. Finally, group comparisons can be influenced by individual differences within the bilingual group. Indeed, exploratory analyses suggested bilingual participants varied in their daily-life use of English or German for number processing, which may impact which language they used during the task. Furthermore, even though all task instructions were provided in German only, with this being an online study, we could not control the language environment people completed the study in. Therefore, it is likely that some bilinguals completed the task in an English environment and some in a more German environment, thereby further increasing the difficulty of seeing any group-level effects.

## Experiment 2

In Experiment 2, we therefore examined the potential influence of language by manipulating language activation *within participants*, rather than relying on group comparisons. This avoided the need for monolingual participants and issues associated with potential group differences beyond people’s language background. By manipulating language within the task itself, we also aimed to minimise the influence of unknown individual differences in language environment while doing the study. We therefore asked participants to describe a simple scene in English or in German (each language used for half of the trials, in response to a language cue) before each number trial within the task. The use of English or German while describing the scene was anticipated to increase the activation of that language immediately prior to making the number-compatibility decision.

Following Grosjean’s Language Mode (1985)^53^, language activation falls on a continuum from predominant activation of one language to activation of multiple languages, depending on the context, conversation partners, and general environment. This shows that language is flexible and can change quickly in response to task context and demands. Many language switching studies have used designs similar to ours, asking participants to name pictures or describe scenes in response to a language cue. These studies typically show switching costs, with participants requiring more time to name a picture when the previous picture required use of another language, compared to when the same language can be used on two consecutive pictures (e.g^54^.,; see^55^, for a review). These costs are usually interpreted as (at least partly) reflecting ongoing competition between languages, with switch trials requiring bilinguals to rapidly re-balance the activation levels of each language.

Further research has also shown that short exposure to a language context (either linguistic or non-linguistic), manipulated on a trial-by-trial basis, can indeed influence activation of the corresponding language. For example, in a simple picture naming task, de Bruin and Martin (2022)^56^showed Basque-Spanish participants linguistic (short sentences) and non-linguistic (flags associated with a language/culture) cues. Participants could name each picture in their language of choice and were free to ignore the “cue”. Nevertheless, participants were more likely to name the picture in the language matching the preceding cue, and were faster naming a picture when following the language indicated by that context, showing fast re-balancing of language activation and use. Similarly, trial-by-trial manipulations of language context have been shown to influence other cognitive processes. For example, interleaving a flanker task with words presented in one or two languages showed an influence of this briefly presented language context on interference suppression (e.g.^57,58^,). This suggests brief exposure to language contexts varying in the activation levels of one or two languages can influence performance on other cognitive tasks.

Indeed, preceding language context has also been shown to influence arithmetic problem solving. Van Rinsveld et al. (2016)^59^ presented multilinguals with arithmetic problems presented without context, or with each number trial preceded by a semantic judgement task that had to be completed in German or French (depending on the language of the session). Complex additions were completed faster in a language context than without context, when the task was completed in French (the participants’ less dominant language). This suggests that manipulating language activation levels throughout the task can indeed influence number processing. In Experiment 2, we therefore expected a larger compatibility effect for number comparison trials following activation of German in the preceding scene description than following activation of English.

## Methods

### Participants

Experiment 2 was completed by 39 German-English bilinguals (*M age* = 29.4 years old, *SD* = 6.8; 21 female, 18 male; 35 right-handed). The mean number of years of education was 17.0 (*SD* = 3.5), with 8 participants completing secondary school/A-levels or lower; 17 an undergraduate degree; 11 a postgraduate degree; and 3 a doctorate. All participants provided informed consent at the start of the study and ethics approval was granted by the Ethics Committee in the Department of Psychology at the University of York. The study was *pre-registered*https://osf.io/nkb96 and was conducted following the Declaration of Helsinki.

Before completing the main study, participants completed a pre-screening. This was completed by 118 potential participants. We only invited participants to take part in the main study if they met the criteria described below. Firstly, invited participants had no neurological, language, or reading difficulties, vision or hearing impairments, or colour blindness. One participant was invited despite responding “yes” to one of these questions and was excluded from analysis (thereby leaving a final sample size of 39 rather than 40). All included participants acquired German from birth and reported not being fluent in any other languages apart from German or English, or the use of any other languages during childhood or current life (i.e., none of the participants reported a language other than German or English in any of the LSBQ questions).

We pre-registered strict inclusion criteria regarding the use of English, including the requirement for participants to be living in an English-speaking country, for them to not select an option lower than “frequently” for weekly English use, or to have an average score below 2.5 across the LSBQ. However, while these requirements were necessary for Experiment 1, they were deemed less essential for Experiment 2, where language was manipulated within participants through the experimental manipulation. We therefore also included participants who used English less frequently than pre-registered. Similar to Experiment 1, we also included two participants with slightly more frequent English use during childhood, as this reflected English use as a teenager. As shown in Table 3, participants had a high proficiency in both languages, as measured through both self-reports and the LexTALE vocabulary scores. All participants, as pre-registered, self-reported their German proficiency as at least 8/10 and their English proficiency as at least 6/10, and had a LexTALE score above 60% in both languages. Participants predominantly used German during their childhood, but currently used English more regularly (all participants reported at least using English sometimes). Participants on average also reported occasional to regular language switching (on a scale from 1 = never to 7 = very frequently; note that this scale differs from Experiment 1) on a daily basis (*M* = 4.7, *SD* = 1.8), within a conversation (*M* = 3.4, *SD* = 1.7), and within a sentence (*M* = 3.0, *SD* = 1.9).

All participants learnt mathematics in German during elementary school and (with the exception of one, who reported using English) during secondary school, and all first learnt numbers in German. Where participants discussed mathematics related topics at University, 26 did so in German and 5 in English. At elementary school, most participants reported studying mathematics for 2–3 h (16) or 3 + hours (19; 4 participants reported 1–2 h). In secondary school, most participants (29) reported 3 + hours of mathematics education (9 2–3 h; 1 1–2 h). Twenty-one participants reported studying additional subjects related to mathematics (e.g., statistics) at school; 19 reported studying a mathematics-related degree at university; and 24 reported using numbers in their jobs. In terms of current use, 35 participants reported using German for tasks such as subtractions (3 reported English and 1 both); 37 participants reported using German for remembering long strings of digits such as phone numbers (2 reported using English). Apart from one person residing in the UK, all participants were living in Germany or Austria. No participants reported learning mathematics, being taught in, or working with numbers in a language other than English or German.

**Table 3:** Participants’ language background in terms of age of acquisition, self-rated proficiency, vocabulary assessed through the LexTALE, and language use.

	Bilinguals
*N*	39
**Age of Acquisition (in years)**	
German	0.2 (1.0)*
English	7.6 (2.4)
**Self-rated German proficiency (1–10)**	
Speaking	10.0 (0.0)
Understanding	10.0 (0.2)
Reading	9.9 (0.2)
Writing	9.9 (0.3)
**Self-rated English proficiency (1–10)**	
Speaking	8.2 (1.1)
Understanding	9.0 (0.9)
Reading	8.9 (1.0)
Writing	8.2 (1.2)
**LexTALE (0–100%)**	
German	89.7 (4.9)
English	84.6 (9.0)
**Language use**	
***L2-English***	
Never	0
Rarely	0
Sometimes	7 people
Frequently	25 people
Always	7 people
**Childhood LSBQ (1 = all German**,** 5 = all English)**	1.7 (0.4)
**Current LSBQ (1 = all German**,** 5 = all English)**	2.3 (0.6)

*Note that one participant reported a German AoA of 6 years old. However, no other languages were reported during childhood or adulthood and other German scores were in line with other participants; the participant was therefore included in the analysis.

Note: The standard deviation is reported in brackets.

### Design

The design was similar to Experiment 1, with one change: Language was manipulated within participants (the scene before each number trial was described in German or in English), rather than manipulated at the group level between participants.

### Procedure and materials

Experiment 2 was run in the same way as Experiment 1, with participants completing a pre-screening questionnaire and the LexTALE before being invited to complete the main task. The main task was the same number magnitude comparison task as in Experiment 1. However, this time, participants were asked to describe a simple scene in German or in English prior to each number magnitude comparison. Half of the scenes had to be described in German and half in English. Participants completed 48 compatible number comparisons (24 preceded by each language); 48 incompatible comparisons (24 preceded by each language); and 24 filler trials (12 per language). Half of the scene descriptions were switch trials (different language had to be used than in the previous scene) and half were non-switch trials (same language had to be used as in the previous scene). These were distributed equally across the compatible and incompatible number trials. Each scene was named five times by each participant, twice preceding a compatible and twice preceding an incompatible trial, and once preceding a filler trial. Two lists were used to counterbalance the number of times each scene was described in German or English (two or three times per language) across participants. Each number pair was presented twice in the main task. With the exception of four pairs, each number pair was preceded once by English and once by a German scene description.

For the scene description task, we selected 25 scenes from previous papers^60,61^. Scenes were selected that could be easily described in one sentence, in German or English. Due to the lexical similarities between the two languages, cognates (words overlapping in form and meaning) could not be avoided.

Prior to starting the task, participants saw an example of a scene and an example of a number comparison trial. Participants were instructed to describe the scenes in a simple way, using a subject-verb-object order to ensure consistency between languages. They were then asked to describe three practice scenes. Following this, they completed three practice number comparison trials, during which they received feedback on their accuracy. Finally, participants completed three practice trials during which they alternated between the scene description and the number comparison trials, always starting with the scene description as in the main task. The main task consisted of six blocks, separated by breaks. The first trial after a break was always a filler trial.

On each trial, the language cue (German or British flag) was first shown in the centre of the screen for 500ms. This was followed by the presentation of the scene. Participants were given five seconds to describe the scene, after which the trial automatically continued. After showing a blank (black) screen for 200ms, the number comparison was presented. This stayed on the screen until a response was given, or for 3000ms if no response was given.

### Data analysis

The task data are available at https://osf.io/gjnwt/.

As first checks, we examined accuracy in the number task and in the scene description task. Accuracy in the main number task was high (above 91% for all participants, *M* = 97.0%, *SD* = 2.4%; across compatible and incompatible trials). Similarly, accuracy in the scene description task across all number trials was high in both L1-German (*M* = 96.6%, *SD* = 3.5%; all participants over 86% correct) and L2-English (*M* = 97.3%, *SD* = 3.3%; all participants over 86% correct; recordings were not audible and therefore not scored for three participants, who were still included in the main analysis). These accuracy scores show that participants indeed paid attention to the task and activated the intended language on each trial. We also computed participants’ naming onset times for each scene (i.e., the delay between the start of scene presentation and the start of naming, using CheckVocal^62)^. We then computed the naming onset time difference between switch trials (trial requiring naming in a different language as the previous trial) and non-switch trials (trial requiring naming in the same language as the previous trial). We did this for English and German separately (e.g., the English switch cost would be the naming time difference between a switch from German to English, and naming two trials in a row in English). As expected, a switch cost was observed in both languages (*M English switch cost* = 43ms, *SD* = 150ms, *M German switch cost* = 41ms, *SD* = 120; including trials included in the main number analysis), suggesting the languages were activated as intended during the scene-description trials and participants had to re-balance language activation during the scene descriptions when switching to a new language.

The main-task analyses were conducted similarly to Experiment 1. 0.5% of correct trials were removed as RT outliers. The RT analysis only included correctly answered number trials that were preceded by a correctly described scene. Type (compatible = −0.5; incompatible = 0.5) and Language (German = −0.5; English = 0.5) were included as fixed effects. Participant, scene picture name, and number item (number pair shown on screen) were included as random effects. Slopes were included for Language and Type for participant and scene picture-name random effects. However, given that a few items did not occur across both languages, no random slopes were included for the number item random effect. The initial model did not converge. We therefore first removed correlations between slopes and intercepts and then (following the pre-registration), the scene picture-name slope explaining the lowest amount of variance. The final model included all random intercepts, all slopes for the participant random effect, and the Type + Type x language slope for the picture-naming random effect. Note that we used the converging model-structure from the LME in the Bayesian model too (for ease of comparison), but additional Bayesian models using either minimal or maximal random-effect structures showed the same results.

## Results and discussion

**Fig. 3:**
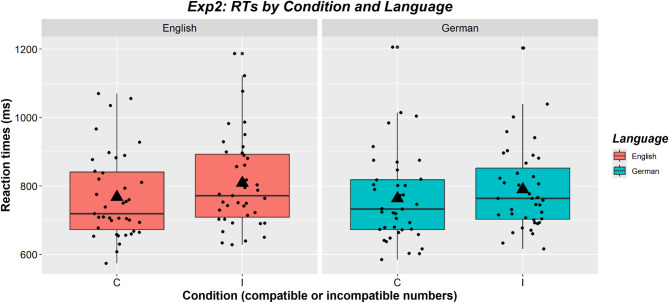
Mean RTs by Type (the condition being Compatible or Incompatible numbers) and Language (number trial preceded by English or German scene description). The centre of the triangle depicts the group mean, the black horizontal line the median. Black dots represent individual participants.

The LME analysis showed that the Type effect did not reach significance (β = 0.047, *SE* = 0.030, *t* = 1.551, *p* =.128), although the numerical pattern went in the expected direction with somewhat faster responses to compatible number pairs (*M* = 766ms, *SD* = 126ms) than to incompatible pairs (*M* = 799ms, *SD* = 126ms), see Fig. 3. There was no main effect of Language (β = 0.012, *SE* = 0.008, *t* = 1.511, *p* =.139), suggesting naming the scene in L1-German or L2-English did not influence RTs on the number-comparison task (after L1-German naming: *M* = 777ms, *SD* = 125ms; after L2-English naming: *M* = 788ms, *SD* = 129ms). The number compatibility effect was numerically a little smaller for after naming in the L1-German (*M* = 26ms, *SD* = 48ms) than after L2-English naming (*M* = 41ms, *SD* = 46ms) but this did not reach significance (β = 0.017, *SE* = 0.014, *t* = 1.287, *p* =.211). If anything, the observed pattern went in the opposite direction than expected (i.e., a slightly smaller compatibility effect after German use, than after English use). The Bayesian model showed a point estimate for the interaction of 0.02, with a 95% credible interval that included 0: [−0.01, 0.05].

The ANOVA showed the same patterns, but the main effect of Trial Type did reach significance (*F*(1,38) = 47.710, *p* <.001, ηp² = 0.557). Neither Language (*F*(1,38) = 2.555, *p* =.118, ηp² = 0.063) nor the interaction between Language and Type reached significance (*F*(1,38) = 1.978, *p* =.168, ηp² = 0.049). The Bayesian ANOVA provided no strong evidence in either direction (i.e., while it trended in the direction of supporting the null hypothesis, it did not clearly support either the presence or absence of a language effect on the number-compatibility effect, BF_01_ = 2.45, error% = 1.03).

One further exploratory analysis was conducted (using LME analyses). If language activation takes time to build up or strengthens with time, we might see a larger effect of naming language on the number task when the same language has been used for at least two scenes. We therefore ran an additional analysis including language trial type (switch/non-switch, with non-switch trials reflecting that the scene was named in the same language as the previous scene). Neither Language Trial type nor its interactions were significant in terms of number-task RTs (p[min] = 0.25). This suggests any influence of language on the number task did not increase when the same language was used multiple times in a row and potentially resulted in stronger activation of that language.

### General discussion

In this paper we were interested in the effect a bilingual’s two languages can have on their processing of (non-linguistic) Arabic digits. We tested L1-German speakers because the compatibility effect in number comparison of Arabic double-digit numbers is significantly larger in participants with a language background that has inverted number words (e.g. German) than in participants whose L1 has (mostly) non-inverted number words (e.g. English). For the same reason, we chose bilingual participants with English as L2.

In Experiment 1, both German-monolinguals and German-English bilinguals showed a significant compatibility effect with longer response times to unit-decade incompatible pairs of Arabic digits. While, as expected, the compatibility effect was numerically smaller in the bilingual group (by 10ms), overall, contrary to our prediction, there was no significant difference in the size of the compatibility effect between the two groups (with some support from the Bayesian analyses for the null). Previous cross-linguistic studies have reported similar, small mean increases in the size of the compatibility effect for adults from a language background with inverted number words compared to those from a language background with non-inverted number words (e.g., 7 ms ^2^, 11 ms^29^, 13 ms^28^). In contrast to our results in Experiment 1 (ηp² = 0.05), in those studies the differences reached significance (with ηp² between 0.09^28^ and 0.11^29^). As discussed later in more detail, there might have been larger inter-individual differences in language activation in our bilingual group than in the monolingual groups tested in previous studies. Also, it could be argued that a smaller effect size would be expected when comparing a monolingual and a bilingual group than when comparing two monolingual groups and that with a larger sample we might have found significant differences in the compatibility effect between the groups.

Between-group comparisons can be hampered by group differences beyond the main factor of interest (here L2). Thus, Experiment 2 employed a within-participant manipulation to avoid potential group differences. The same number comparison task was used with German-English bilinguals, but now participants described scenes in either English or German before each number-comparison trial. While participants, as intended, activated the required language on each trial (and showed a switching cost reflecting re-balancing that language activation), there was no significant difference (with some support from the Bayesian analyses) in the size of the compatibility effect when participants had just activated German versus when they had just activated English. The compatibility effect as such only reached significance in the ANOVA. This is potentially due to the LME analysis capturing more variability related to both number-items and picture-naming-items (cf. also Nuerk et al., 2005^28^, showing a significant compatibility effect in by-participant but not in by-item analyses). Regardless, none of the analyses suggested a significant impact of language. Taken together, our findings suggest that L2-English use in German-English bilinguals has little influence on number comparison with Arabic digits.

Previous evidence showed that the size of the compatibility effect depends on the L1 spoken^28,29^.This strongly suggests that number comparison of double-digit Arabic numbers often activates the indirect processing path, with the verbal code used depending on the languages spoken. The Bilingual Triple Code Model (BTCM^37)^ proposed that in bilinguals, a second indirect path exists in the form of a verbal L2 code. Our results provide no direct evidence for the activation of a second indirect, verbal L2 code, path between the visual and approximate number codes. There are various reasons that can explain these findings.

First, findings from number word priming in bilinguals (e.g.^37,63^) suggest the verbal L2 code pathway might be weaker (or even absent) than the proposed L1 code pathway in the BTCM. For example, Lachelin et al. (2024)^37^showed the priming distance effect in proficient German-French bilinguals was weaker in French (L2) than in German (L1), thus emphasizing a stronger reliance on the verbal L1 code even when L2 number words were used as primes. Their bilingual participants grew up speaking Luxembourgish at home (Home language), learnt German (L1 in their study) early and had numeracy instruction in German at school from grade 1–6 and then in French from grade 7 onwards. Lachelin et al. therefore suggested that the earlier L1 than L2 age of acquisition, as well as the closer language proximity between L1-German and their Home language, might have resulted in a weaker activation of the verbal L2 code, compared to the L1. Other studies also report language of schooling as a strong predictor of Arabic digit naming (e.g.^64^).

In line with this, our bilingual participants all had an earlier age of acquisition for German (L1) than English (L2), acquired number words first in German (L1) and had most of their schooling in German (L1). It is therefore possible that for these bilinguals, the verbal L1 code was so strongly established from a young age that all number processing continues to go through the verbal L1 code (rather than, or to a much lesser extent, the L2 code). Further research is therefore needed to better understand the potential role of the language of schooling in bilinguals living in bilingual societies as well as in bilinguals who grew up in a different language environment than the one they are currently living in (as was the case for many of our bilinguals). Further research will also need to further study the role of current daily-life language use in relation to number processing. Our exploratory analyses in Experiment 1 did not show a significant relationship between the compatibility effect and the participant’s daily-life language use for number processing, potentially due to the small sample size and the lack of a detailed questionnaire about current language use for number processing. However, the numerical pattern suggested further research is warranted to better understand a possible effect of daily-life language use for number processing (as well as other individual differences in, for example, proficiency and number-processing strategies) on the activation of an indirect route through the L1 or L2.

Participants in Experiment 1 might have differed in the extent to which they activated the indirect route and possibly the verbal L2 code. We therefore manipulated L1 or L2 activation directly in Experiment 2. Despite the language manipulation “working” as intended (i.e., high accuracy and switching costs), there was no evidence for the activation of the second indirect path between the visual and approximate number codes via the verbal L2 code during number comparison, even when participants had just described a visual scene in L2. This argues further for a preference of L1 for number processing in our bilinguals, which might have been so strong that this was not overruled by a temporary boost of L2.

A second explanation, specifically concerning Experiment 2, is that perhaps a longer L2 boost is needed for the indirect path through the verbal L2 code to be used. In our experiment, language activation was manipulated on a trial-by-trial level. While this has successfully been shown to influence language-related mechanisms in previous literature (e.g.^56^), such a short-lived activation change might not be sufficient to influence other cognitive processes. Indeed, Van Rinsveld and colleagues (2016)^59^ showed an impact of language context on arithmetic tasks but manipulated language through longer contextual blocks. While exploratory analyses suggested that the additional time of using the L2 (through multiple consecutive scene descriptions in that language) did not increase its influence on the number processing task, it remains possible that such L2 effects only arise after longer (e.g., at least 5–10 min) blocks of L2 use.

Finally, a third potential explanation for our findings could be that participants in Experiment 1 did not activate number words at all while performing the number comparison task with Arabic digits. This is in contrast to findings from several other studies with monolinguals^28,29^, but in line with findings from Macizo et al. (2011)^39^. They compared the compatibility effect between Spanish-English bilinguals who learnt number words first in Spanish and German-English bilinguals who learnt number words first in German. There were no significant differences between the two bilingual groups in the size of their compatibility effect for Arabic digits. If their bilinguals had activated their indirect path via the verbal L1 code, the size of the compatibility effect should have differed between these two groups. Therefore, a potential interpretation of our findings remains that participants did not use number word activation during the task. It is also possible that interindividual differences in the extent to which our participants activated number words could have reduced the overall compatibility effect and diminished any L1-L2 differences. In addition, in Experiment 2 we reduced the number of items to 96. This was necessary to allow enough time in the experiment for the scene descriptions before each item, but this could also have affected our ability to detect a group difference in the compatibility effect.

Our findings are in contrast to findings on the influence of spatial reference frames on number processing. In a previous study, Moeller et al. (2015)^2^showed that both number word inversion as well as habitual reading/writing direction (left-to-right vs. right-to-left) can influence the compatibility effect in symbolic number comparison. Interestingly, and in contrast to our findings, when spatial reference frames were changed in bilingual participants, it influenced their number processing, e.g.^65,66^. This is in line with other findings^67–70^ and highlights a clear difference between the influence of spatial versus linguistic context on symbolic number processing. While spatial reference frames are constructed temporarily and their influence on number representations seems to be flexible and context-dependent in bilinguals, the linguistic influence of the L1, be it because of the earlier onset of acquisition or because it was the language of (mathematical) instruction, seems to have a more permanent dominant effect on symbolic number processing in bilinguals. Future studies need to test this conclusion more robustly by, for example, manipulating the extent of bilinguals’ current use of number words in L2.

## Data Availability

The main task data are available at https://osf.io/gjnwt/.
